# Adipocyte NADH dehydrogenase reverses circadian and diet-induced metabolic syndrome

**DOI:** 10.1038/s42255-026-01464-5

**Published:** 2026-02-18

**Authors:** Chelsea Hepler, Nathan J. Waldeck, Benjamin J. Weidemann, Biliana Marcheva, You-Jia Chen, Jacqueline Hecker, Ziming Zhu, Rino Nozawa, Joseph V. Mastroni, Anneke K. Thorne, Colleen R. Reczek, Jonathan Cedernaes, Kathryn M. Ramsey, Clara B. Peek, Grant D. Barish, Navdeep S. Chandel, Joseph Bass

**Affiliations:** 1 Department of Medicine, Division of Endocrinology, Metabolism, and Molecular Medicine, Feinberg School of Medicine, Northwestern University, Chicago, IL USA; 2 Department of Molecular & Integrative Physiology, University of Michigan, Ann Arbor, MI USA; 3 Division of Pulmonary and Critical Care Medicine, Department of Medicine, Northwestern University, Chicago, IL USA; 4 Department of Biochemistry and Molecular Genetics, Northwestern University, Chicago, IL USA

**Keywords:** Circadian rhythms, Energy metabolism, Metabolism

## Abstract

Circadian clocks are internal timing systems that enable organisms to anticipate and adapt to daily environmental changes. These rhythms arise from a transcription–translation feedback loop in which CLOCK and BMAL1 regulate the expression of thousands of genes, including their repressors PER and CRY. Disruption of circadian rhythms contributes to obesity, metabolic disease and cancer, yet how the clock maintains metabolic homeostasis remains limited. Here we report that the clock regulates oxidative metabolism in adipocytes through diurnal complex I respiration. Disrupting the clock in male mice via adipocyte-specific genetic deletion or high-fat-diet feeding reduces complex I respiration in adipocytes, leading to suppression of the peroxisome proliferator-activated receptor and insulin signalling pathways. In contrast, restoring complex I function by expressing yeast NDI1 in adipocytes protects against diet-induced and circadian-induced metabolic dysfunction independently of weight gain. These findings reveal that adipocyte circadian disruption impairs metabolic health through mitochondrial complex I dysfunction, establishing clock control of complex I as a key regulator of metabolic homeostasis.

## Abstract (web summary)

Circadian clock in adipocytes maintains metabolic health by regulating mitochondrial complex I respiration.

## Main

High-fat feeding leads to circadian disruption^1^, suggesting that an understanding of how clock transcription cycles control physiology will elucidate general mechanisms of metabolic disease. At the molecular level, the core clock transcription–translation feedback loop is driven by the activators CLOCK and BMAL1, which induce the expression of the repressors PER and CRY1 (ref. ^1^). In turn, PER and CRY bind to CLOCK and BMAL1 to inhibit their own expression. While there is extensive knowledge of the interplay of core clock proteins in the central control of the sleep–wake cycle, our understanding of how peripheral clocks regulate metabolism and health remains limited.

The circadian clock has a pivotal role in physiological rhythms through direct transcriptional control of rate-limiting metabolic enzymes^2,3^ and indirectly through epigenetic pathways that direct fuel switching between oxidative and reductive metabolism across the fasting and feeding cycle. For example, BMAL1 directly controls transcription of enzymes involved in NAD^+^ biosynthesis^4^. This in turn drives rhythms in fatty acid oxidation through time-dependent activation of NAD^+^-dependent enzymes, including sirtuins and poly (ADP-ribose) polymerases^5,6^. Peripheral clocks also exhibit distinct tissue-specific properties; for instance, the liver clock is entrained by feeding time, whereas the white adipose tissue clock aligns with the light–dark cycle^1,7–9^.

Obesity is associated with pronounced disruption in circadian clock gene expression within adipose tissue^1,10^. Mice with genetic ablation of the adipocyte clock display enhanced weight gain during high-fat-diet (HFD) feeding, increased visceral adiposity and arrhythmic food intake with increased light-phase feeding^11^. These findings reveal that circadian disruption in adipocytes contributes to systemic metabolic disorders during overnutrition. Our prior work revealed a role for the adipocyte circadian clock in regulating energy balance according to the time when an animal eats through control of creatine-mediated thermogenesis^8^. In this study, we sought to determine how genetic ablation of the circadian clock in adipocytes exerts effects beyond the dysregulation of diet-induced thermogenesis and creatine cycling^12,13^.

**Fig. 1:**
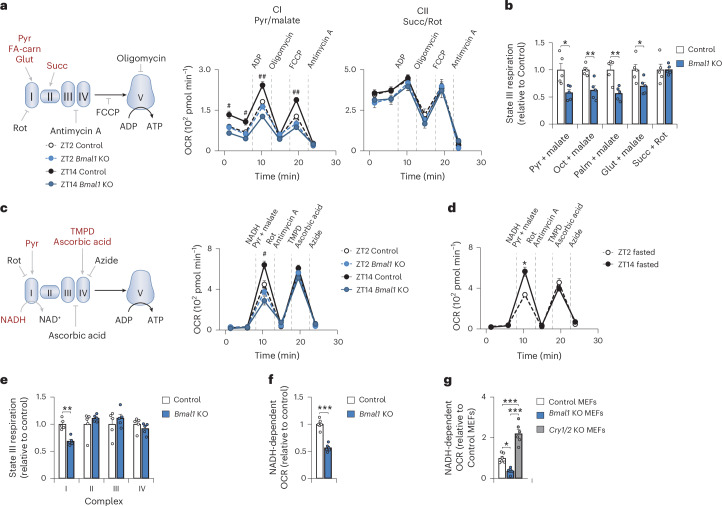
The circadian clock regulates respiration at the mitochondrial respiratory chain CI. **a**, OCR in gWAT mitochondria isolated from control (*Bmal1*^*loxP/loxP*^) and *Bmal1* KO (*Adipoq-cre; Bmal1*^*loxP/loxP*^) mice at ZT2 and ZT14 in the presence of the indicated substrates and inhibitors (*n* = 7 control and *n* = 6 *Bmal1* KO for pyruvate (Pyr), and *n* = 6–7 control and *n* = 5 for *Bmal1* KO for succinate (Succ)). **b**, State III respiration in gWAT mitochondria isolated from control and *Bmal1* KO mice at ZT14 in response to CI-linked and CII-linked substrates (*n* = 5). **c**, OCR in gWAT mitochondria of control and *Bmal1* KO mice at ZT2 and ZT14 in the presence of the indicated substrates and inhibitors (*n* = 5). **d**, OCR in gWAT mitochondria of wild-type mice at ZT2 and ZT14 after 12 h of fasting in response to the indicated substances as in **c** (*n* = 5). **e**, State III respiration in adipocytes differentiated from control and *Bmal1* KO mice in the presence of the CI–IV substrates (*n* = 5). **f**, NADH-dependent OCR in adipocytes differentiated from control and *Bmal1* KO mice (*n* = 6). **g**, NADH-dependent OCR in control, *Bmal1*^−/−^ and *Cry1/2*^−/−^ MEFs (*n* = 6). Data are presented as the mean ± s.e.m. **a**,**c**, Statistical significance was calculated using a two-way analysis of variance (ANOVA) followed by Dunnett’s multiple comparisons test with the ZT14 control set as the reference group. **b**,**e**, A multiple unpaired two-sided *t*-test was used. **d**, A two-way ANOVA followed by multiple comparisons test was used. **f**, An unpaired two-sided *t*-test was used. **g**, A one-way ANOVA followed by multiple comparisons test was used. The # symbol denotes the statistical significance between ZT14 control and all other groups. *P < 0.05, **P < 0.01, ***P < 0.001. Source data

To determine how the circadian clock affects mitochondrial metabolism in adipose tissue, we isolated mitochondria from the gonadal white adipose tissue (gWAT) of male mice entrained to a normal or inverted light cycle at different times of the night and day (zeitgeber time (ZT)). The mitochondrial oxygen consumption rate (OCR) in the presence of the complex I (CI)-linked substrate pyruvate was increased during the dark–active period (ZT14) compared with the light/inactive period (ZT2) (Fig. 1a). Mice lacking the clock activator *Bmal1* in adipocytes (*Bmal1* knockout (KO)) displayed reduced mitochondrial OCR in response to pyruvate during the dark period (ZT14) compared with mice with an intact clock. However, mitochondrial OCR in the presence of the complex II (CII)-linked substrate succinate was not different in circadian mutant adipose tissue mitochondria (Fig. 1a). Carbonyl cyanide-*p*-trifluoromethoxyphenylhydrazone (FCCP)-stimulated respiration was lower than ADP-stimulated respiration, a pattern observed when FCCP or substrates become limiting and previously reported in Seahorse assays using isolated mitochondria^6,14^. Collectively, our results show that adipocyte CI respiration but not CII respiration is diurnal and dependent on the clock.

**Extended Data Fig. 1:**
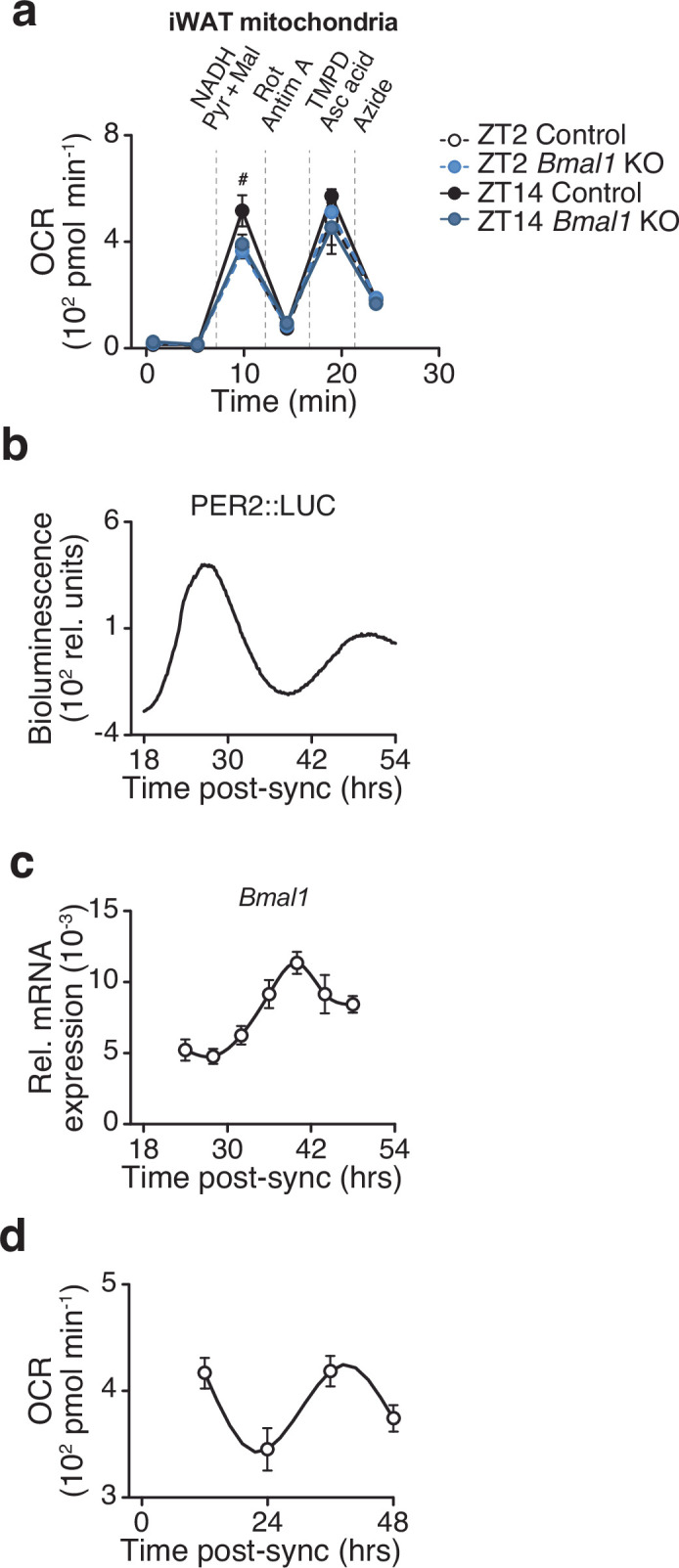
The circadian clock regulates mitochondrial complex I respiration, related to Fig. 1. **a**, OCR in iWAT mitochondria of Control and *Bmal1*-KO mice at ZT2 and ZT14 in the presence of indicated substrates and inhibitors (n = 4). TMPD, Tetramethyl-p-phenylenediamine; Asc acid; ascorbic acid. **b**, Representative bioluminescence of PER2::LUC oscillations in synchronized primary white adipocytes from mPer2:Luc mice. **c**, Relative mRNA expression of Bmal1 in synchronized white adipocytes in vitro (n = 6 per timepoint). **d**, NADH-dependent respiration in synchronized white adipocytes in vitro (n = 6 per timepoint). Data are represented as mean ± SEM. Statistical significance was calculated by two-way ANOVA followed by Dunnett’s multiple comparisons test with ZT14 Control set as the reference group in **a**. Source data

We next assessed respiration in response to distinct substrates that feed into CI via NADH generation from glycolysis, beta oxidation and glutaminolysis. Mitochondria from adipocyte *Bmal1* KO mice had reduced ADP-stimulated (state III) respiration in the presence of NADH-generating substrates, but not succinate (Fig. 1b). To determine whether the respiratory deficit in circadian mutant adipocytes occurs at or upstream of CI, we measured respiration in response to NADH. We found that NADH-fuelled respiration was elevated at ZT14 and reduced by *Bmal1* deletion (Fig. 1c). Similar rhythmic, clock-dependent CI respiration was observed in inguinal WAT (iWAT) (Extended Data Fig. 1a).

To distinguish whether respiration was determined by intrinsic rhythmicity or feeding state, we analysed WAT mitochondria at ZT2 and ZT14 after a 12-h fast. Even under these conditions, NADH-fuelled CI respiration remained higher at ZT14 than ZT2 (Fig. 1d). However, a single overnight fast does not fully eliminate feeding effects. Therefore, to circumvent feeding rhythms altogether, we performed Seahorse assays in cultured adipocytes. In permeabilized adipocytes, OCR in response to CI substrate (pyruvate), but not CII (succinate), CIII (duroquinol) or CIV (tetramethyl-p-phenylenediamine (TMPD)/ascorbate), was reduced in *Bmal1*-deficient cells (Fig. 1e). NADH-fuelled CI respiration was also rhythmic in synchronized adipocytes (Extended Data Fig. 1b–d) and decreased in adipocytes lacking *Bmal1* in vitro (Fig. 1f). Consistent with these results, mouse embryonic fibroblasts (MEFs) lacking the clock activator *Bmal1* displayed reduced CI respiration, whereas MEFs lacking the repressors *Cry1* and *Cry2* exhibited increased CI respiration (Fig. 1g). These data reveal opposing effects of the forward (BMAL1-mediated) and reverse (CRY-mediated) limbs of the intrinsic clock on CI respiration, independent of nutrient availability.

**Extended Data Fig. 2:**
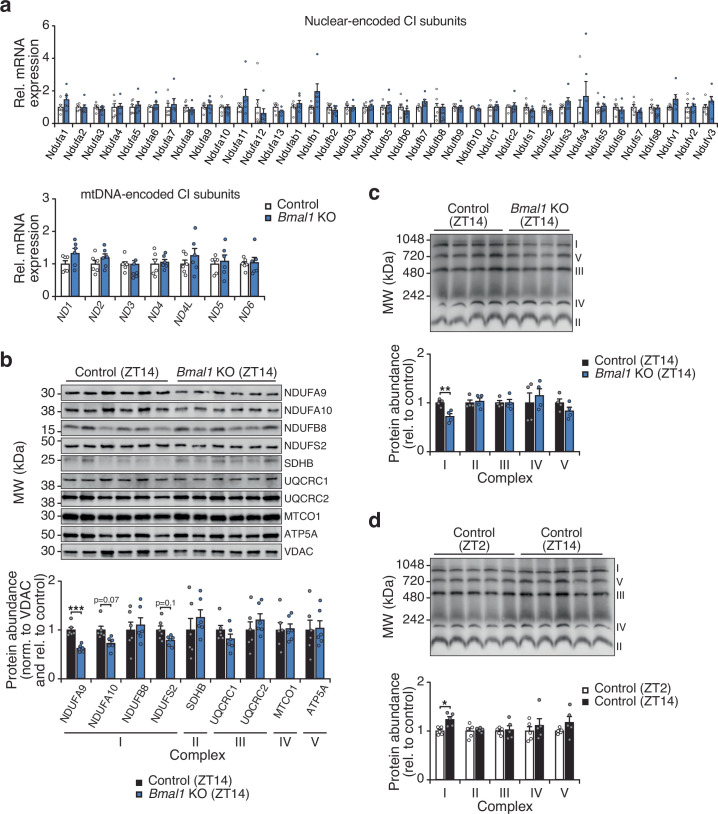
The circadian clock regulates the abundance of mitochondrial complex I, related to Fig. 1. **a**, mRNA abundance of complex I subunits in adipocytes from the gWAT depot of Control (*Bmal1*^*fx/fx*^) and *Bmal1*-KO (*Adiponectin-Cre; Bmal1*^*fx/fx*^) mice (n = 6). **b**, SDS-PAGE immunoblot of electron transport chain proteins in isolated WAT mitochondria from Control and *Bmal1-KO* mice harvested at the indicated timepoints (n = 6). **c**, BN-PAGE immunoblot on isolated WAT mitochondria from Control and *Bmal1-KO* mice at ZT14 using an antibody mix against OXPHOS complex subunits (n = 4). **d**, BN-PAGE immunoblot on isolated WAT mitochondria from Control mice at ZT2 and ZT14 using an antibody mix against OXPHOS complex subunits (n = 5). Data are represented as mean ± SEM. Statistical significance was calculated by two-way ANOVA followed by multiple comparisons in **a-d**. Source data

Mammalian mitochondrial CI is the largest respiratory enzyme, consisting of 45 subunits^15^. The assembly of CI necessitates the precise coordination of several assembly factors as well as all nuclear and mitochondrial DNA-encoded subunits to form a fully operational respiratory complex. This process is regulated both transcriptionally and through posttranslational modifications driven by nutrient and redox status^16^. Ablation of the clock did not affect mRNA abundance of the nuclear or mitochondrial DNA-encoded CI subunits (Extended Data Fig. 2a). However, the protein abundance of the CI subunit NDUFA9 was reduced in mitochondria from adipocyte *Bmal1* KO mice, while NDUFA10 and NDUFS2 were trending lower but did not reach statistical significant (*P* = 0.07 and *P* = 0.1, respectively) (Extended Data Fig. 2b). Consistent with the loss of some protein of the CI subunits, the total abundance of fully assembled CI was reduced in mitochondria from adipocyte *Bmal1* KO mice (Extended Data Fig. 2c). In addition, WAT mitochondria from mice isolated at ZT2 showed a decreased abundance of CI compared with ZT14 (Extended Data Fig. 2d). Neither genetic deletion of the clock nor time of day affected the total amount of CII–V. Together, these results indicate that the abundance of mitochondrial CI is regulated by the circadian clock independently of direct transcriptional control.

**Extended Data Fig. 3:**
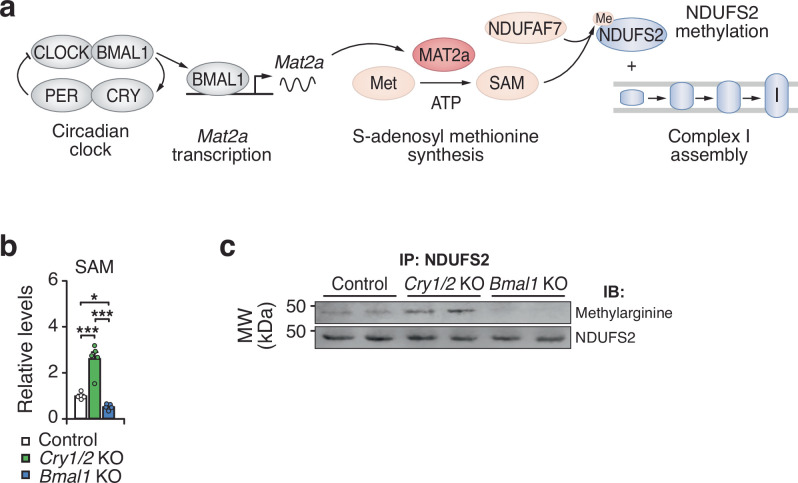
Circadian control of SAM-dependent NDUFS2 methylation, related to Fig. 1. **a**, Model illustrating how BMAL1 regulates methionine metabolism and SAM production through *Mat2a*, enabling NDUFAF7-mediated methylation of NDUFS2 required for proper complex I assembly. **b**, SAM levels in Control, *Bmal1*^*−/−*^ and *Cry1/2*^*−/−*^ MEFs (n = 5 technical replicates per genotype). **c**, Methylarginine and NDUFS2 western blotting of immunoprecipitated NDUFS2 in Control, *Bmal1*^*−/−*^ and *Cry1/2*^*−/−*^ MEFs (n = 2). Data are represented as mean ± SEM. Statistical significance was calculated by one-way ANOVA followed by multiple comparisons in **b**. * p < 0.05, **p < 0.01, ***p < 0.001. Source data

We next investigated a potential posttranscriptional mechanism according to which the clock regulates CI function. *S*-adenosyl methionine (SAM) is the universal methyl donor in energy metabolism and is produced from methionine by the methyltransferase MAT2A. We previously found that BMAL1 binds to the *Mat2a* promoter in adipocytes and regulates its expression, while deletion of Bmal1 reduces both MAT2A protein and SAM abundance^8^. Methylation of the CI subunit NDUFS2 is critical for mitochondrial metabolism; hypomethylation impairs its stability^17–19^. Therefore, we hypothesized that circadian control of SAM availability contributes to CI regulation (Extended Data Fig. 3a). MEFs lacking *Bmal1* showed lower CI respiration, reduced SAM levels and decreased NDUFS2 methylation (Fig. 1g and Extended Data Fig. 3b,c). By contrast, *Cry1*-deficient and *Cr2*-deficient MEFs exhibited increased CI respiration, increased SAM and enhanced NDUFS2 methylation (Fig. 1g and Extended Data Fig. 3b,c). Together, these findings identify SAM-dependent methylation of NDUFS2 as a mechanism that links the circadian clock to CI assembly and activity in adipocytes.

**Extended Data Fig. 4:**
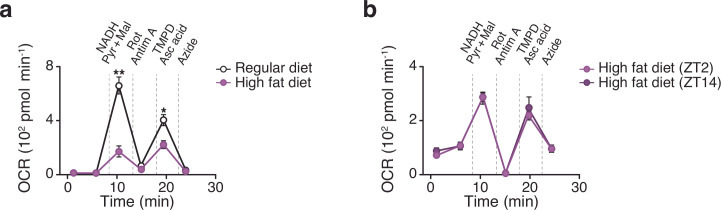
High fat diet feeding leads to reduced and loss of diurnal CI respiration in visceral WAT mitochondria, related to Fig. 2. **a**, OCR in WAT mitochondria of wildtype mice at ZT14 following 12 weeks of chow or HFD feeding. Mitochondria were treated with pyruvate/malate/FCCP followed sequentially with rotenone, succinate, antimycin A, and N,N,N′,N′-Tetramethyl-p-phenylenediamine (TMPD)/ascorbic acid (n = 5). **b**, OCR in WAT mitochondria of wildtype mice at ZT2 and ZT14 following 12 weeks of HFD feeding. Mitochondria were treated with pyruvate/malate/NADH followed sequentially with rotenone/antimycin A, TMPD/ascorbic acid, and azide (n = 6). Data are represented as mean ± SEM. Statistical significance was calculated by two-way ANOVA followed by multiple comparisons in **a-b**. **p < 0.01. Source data

Genetic studies in mice have shown that reduced mitochondrial function in adipocytes has a causative role in systemic insulin resistance^20,21^. Obesity is associated with impaired adipose tissue mitochondrial function and a reduction in circadian clock genes in adipose tissue^1,22,23^. Supporting this finding, we observed reduced and loss of diurnal CI respiration in gWAT mitochondria after HFD feeding (Extended Data Fig. 4a,b). We hypothesized that circadian disruption during obesity impairs mitochondrial function through dysregulation at CI.

**Fig. 2:**
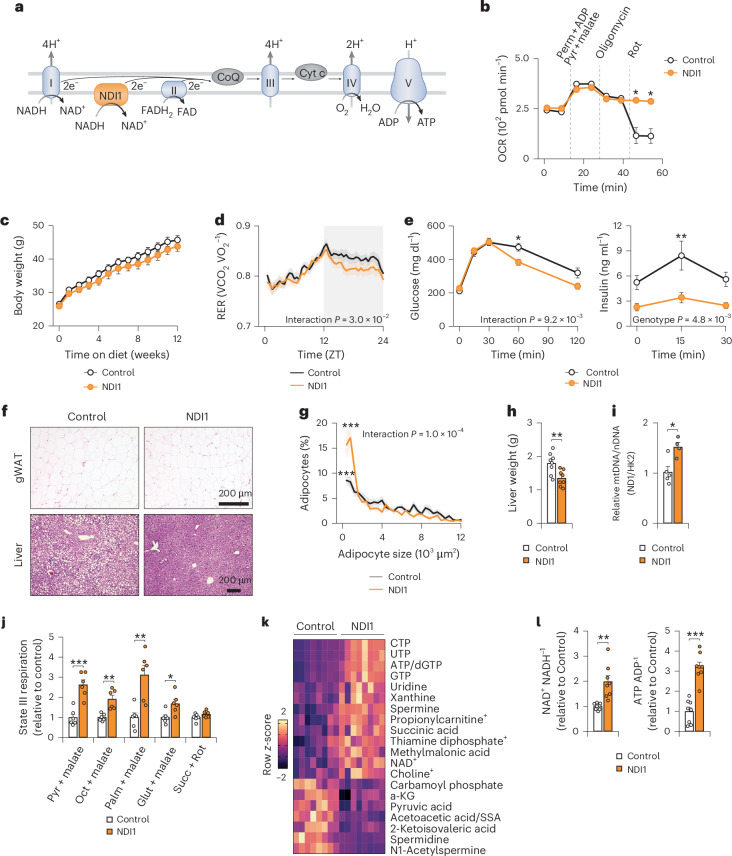
Expression of the yeast NDI1, an alternative NADH dehydrogenase, in adipocytes improves metabolic health during HFD feeding. **a**, Schematic of the mitochondrial ETC with ectopic yeast NDI1. NDI1 transfers electrons to ubiquinone and regenerates NAD^+^. **b**, OCR of adipocytes differentiated from control (*NDI1*^*LSL*^) and NDI1 (*Adipoq-cre; NDI1*^*LSL*^) mice in the presence of the indicated substrates and inhibitors (*n* = 6 control and *n* = 5 NDI1). **c**, Body weight during HFD feeding for 12 weeks in control and NDI1 mice (*n* = 8). **d**, RER over 24 h after 10 weeks of HFD feeding (*n* = 8). **e**, GTT and insulin during the GTT at 12 weeks of HFD feeding (*n* = 8 mice per group). **f**, Representative images of haematoxylin and eosin (H&E) staining of gWAT and liver after 12 weeks of HFD feeding (×20 and ×10 magnification, respectively). **g**, Distribution in gWAT adipocyte size after 12 weeks of HFD feeding (*n* = 5). **h**, Liver weight after 12 weeks of HFD feeding (*n* = 8). **i**, Mitochondrial-to-nuclear DNA ratio in gWAT after 12 weeks of HFD feeding (*n* = 5 control and *n* = 4 NDI1). **j**, State III respiration in gWAT mitochondria in response to CI-linked and CII-linked substrates after 12 weeks of HFD feeding (*n* = 6). **k**, Heatmap of differentially abundant metabolites (*P* < 0.05 and false discovery rate (FDR) < 0.10, calculated using the two-step Benjamini, Krieger and Yekutieli multiple comparison adjustment approach) in gWAT from control and NDI1 mice after 12 weeks of HFD feeding at ZT14 (*n* = 8). **l**, The NAD^+^-to-NADH and ATP-to-ADP ratios in WAT from control and NDI1 mice after 12 weeks of HFD feeding at ZT14 (*n* = 8). Data are presented as the mean ± s.e.m. **b**–**f**,**g**,**j**, Statistical significance was calculated using a two-way ANOVA followed by multiple comparisons. **h**,**i**,**l**, An unpaired two-sided *t*-test was used. *P < 0.05, **P < 0.01, ***P < 0.001. Source data

**Extended Data Fig. 5:**
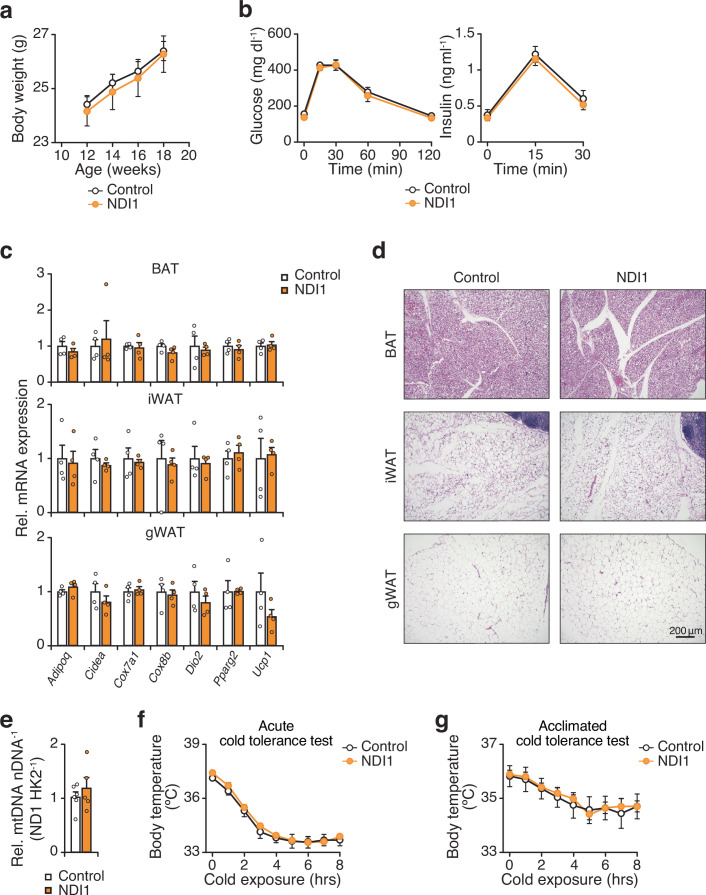
NDI1 expression does not affect adipocyte physiology or glucose homeostasis in chow-fed mice, related to Fig. 2. **a**, Body weight of Control (*NDI1*^*LSL*^) and NDI1 (*Adiponectin-Cre; NDI1*^*LSL*^) mice at their indicated age on a chow diet (n = 6 Control and 5 NDI1). **b**, Glucose tolerance test (GTT) and insulin during the GTT at 3 months of age (n = 6 Control and 5 NDI1). **c**, Gene expression of indicated genes involved in thermogenesis in brown adipose tissue (BAT), inguinal white adipose tissue (iWAT), and gonadal white adipose tissue (gWAT) of Control and NDI1 mice at 3 months of age (n = 4). **d**, Representative images of hematoxylin and eosin staining of indicated adipose tissue depots in Control and NDI1 mice at 3 months of age (10x magnification, scale bars 200 μm). **e**, Mitochondria/nuclear DNA ratio in gWAT from control and NDI1 mice at 3 months of age (n = 6 Control and 5 NDI1). **f**, Body temperature at indicated time points following transfer of mice from room temperature to cold (6 °C) (n = 5 Control and n = 6 NDI1). **g**, Body temperature at indicated time points during a cold tolerance test after 4 weeks of acclimation to cold (6 °C) (n = 5 Control and n = 6 NDI1). Data are represented as mean ± SEM. Statistical significance was calculated by two-way ANOVA followed by multiple comparisons in **a-c, f**, and **g** and unpaired two-sided t-test in **e**. Source data

To investigate the role of adipocyte CI function, we generated mice with conditional expression of yeast NADH dehydrogenase (NDI1)^24,25^ in adipocytes (*Adipoq-cre;NDI1*^*LSL*^). NDI1 is a rotenone (Rot)-insensitive, non-proton-translocating NADH dehydrogenase that regenerates NAD^+^ and transfers electrons to ubiquinone^26^ (Fig. 2a,b). Therefore, NDI1 can restore oxidative phosphorylation through functionally replacing CI, independently of time of day^16,27^. In lean adult mice, the expression of NDI1 in adipocytes had no impact on body weight, glucose homeostasis, the expression of mature adipocyte and thermogenic markers, or histological features of adipose depots (Extended Data Fig. 5a–e). NDI1 expression also did not affect body temperature maintenance during acute and adapted cold tolerance testing (Extended Data Fig. 5f,g).

**Extended Data Fig. 6:**
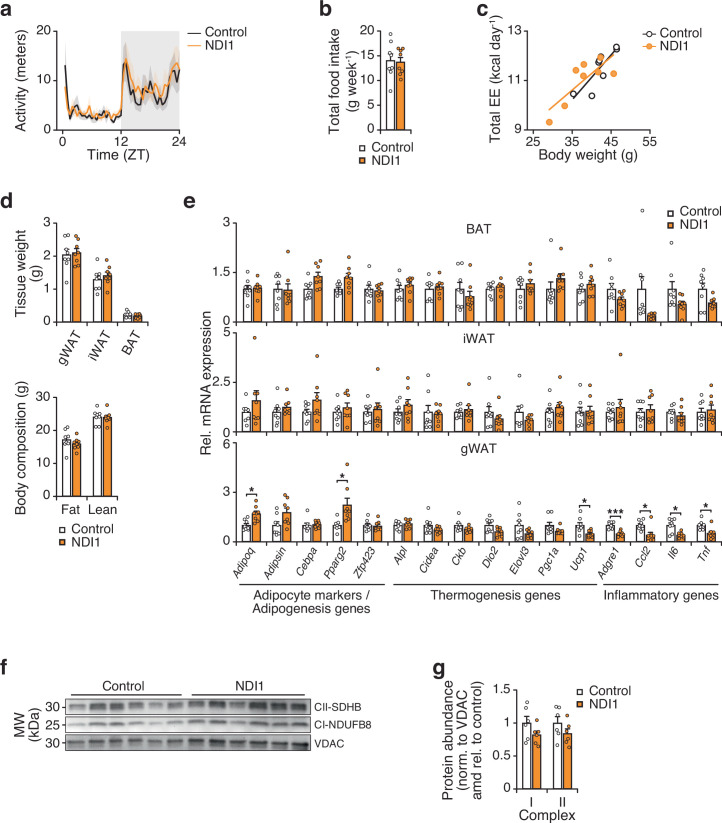
Expression of NDI1 does not affect activity, food intake, or energy expenditure during high fat diet feeding, related to Fig. 2. **a**, Daily activity rhythms of Control and NDI1 mice after 10 weeks of HFD feeding (n = 8). **b**, Total daily food intake in Control and NDI1 mice after 10 weeks of HFD feeding (n = 8). **c**, Total energy expenditure over 24 hours versus body weight after 10 weeks of HFD feeding (n = 8). **d**, Fat pad weights and body composition of Control and NDI1 mice after 12 weeks of HFD feeding (n = 8). **e**, Gene expression of indicated genes involved in thermogenesis in BAT, inguinal WAT, and gWAT from Control and NDI1 mice after 12 weeks of HFD feeding (n = 8). **f**, SDS-PAGE immunoblot of complex I and II subunits in isolated gWAT mitochondria from Control and NDI1 mice after 10 weeks of HFD feeding (n = 6). **g** Mitochondria/nuclear DNA ratio in gWAT from control and NDI1 mice after 10 weeks of HFD feeding (n = 6). Data are represented as mean ± SEM. Statistical significance was calculated by two-way ANOVA followed by multiple comparisons in **a**, **d**, **e**, and **g**, unpaired two-sided t-test in **b** and ANCOVA in **c**. *p < 0.05, ***p < 0.001. Source data

We next tested whether preserving adipocyte mitochondrial CI function during HFD could prevent obesity and metabolic syndrome. Mice expressing adipocyte NDI1 displayed similar weight gain, activity, caloric intake, energy expenditure, fat pad weights and body composition during HFD feeding compared with control mice (Fig. 2c and Extended Data Fig. 6a–d). However, NDI1 expression led to a reduced respiratory exchange ratio (RER) during the dark/active period, indicating a shift towards lipid metabolism (Fig. 2d). Remarkably, NDI1 expression led to improved glucose tolerance and reduced insulin release during the glucose tolerance test (GTT) (Fig. 2e).

Histological analysis revealed smaller adipocytes in gWAT and reduced hepatic steatosis in mice expressing NDI1 in adipocytes (Fig. 2f–h). NDI1 expression did not have any major effects on gene expression in brown adipose fat (BAT) and iWAT (Extended Data Fig. 6e). However, NDI1 expression increased adipogenic markers (*Pparg2* and *Adipoq*) and decreased several inflammatory markers (*Adgre1*, *Ccl2*, *Il6* and *Tnf*) in gWAT (Extended Data Fig. 6e). This gene expression profile, along with the smaller visceral adipocyte size, is consistent with healthy visceral WAT remodelling during HFD feeding in NDI1-expressing mice. gWAT from NDI1-expressing mice exhibited increased mitochondrial DNA content and enhanced CI-driven but not CII-driven respiration, without changes in the abundance of either complex (Fig. 2i,j and Extended Data Fig. 6f,g). Metabolomics of gWAT revealed that NDI1 expression led to an increase in abundance of several nucleotides (NAD^+^, ATP, GTP) and succinic acid, a reduction in pyruvic acid and α-ketoglutarate, and an increase in the NAD^+^-to-NADH and ATP-to-ADP ratios (Fig. 2k,l). Thus, preserving adipocyte CI function with NDI1 sustained NADH oxidation and mitochondrial respiration, enabling visceral WAT to remodel through hyperplasia rather than hypertrophy during HFD feeding. Collectively, these findings indicate that reduced adipocyte mitochondrial CI activity during obesity is a key driver of visceral WAT metabolic dysfunction and subsequent systemic impairment in metabolic health.

The improved gene expression profile in visceral WAT in NDI1-expressing mice after HFD feeding prompted us to further examine the role of CI in visceral adipocytes. Defects in CI are among the most common causes of mitochondrial disorders and have been linked to a wide range of pathologies, including neurodegenerative and cardiac diseases^28^. Genetic CI dysfunction in several tissues remodels the global transcriptome and alters cell state through retrograde signalling^24,25^. To investigate how CI deficiency affects adipocyte biology, we generated mice lacking the core catalytic subunit of CI, *Ndufs2*, specifically within adipocytes^29^. Loss of NDUFS2 leads to complete CI deficiency because *NDUFS2* encodes an essential catalytic core subunit required for enzyme assembly and electron transfer^30^.

**Fig. 3:**
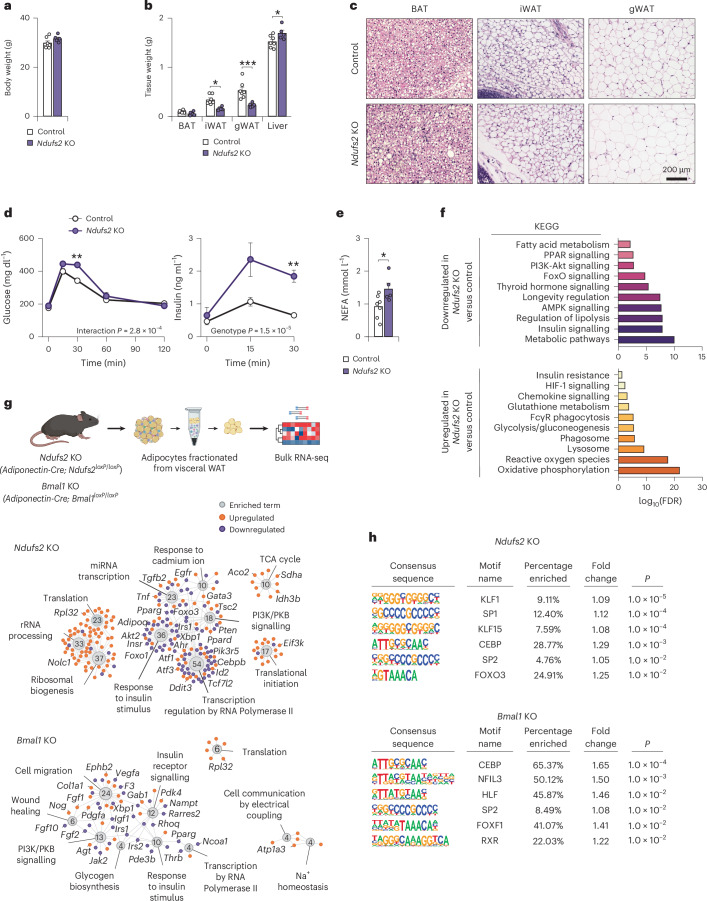
Adipocyte CI deficiency limits adipose expansion and impairs systemic glucose homeostasis. **a**, Body weight of control (*Ndufs2*^*loxP/loxP*^) and *Ndufs2* KO (*Adipoq-cre; Ndufs2*^*loxP/loxP*^) mice at 4 months of age on a chow diet (*n* = 7 control and *n* = 5 *Ndufs2* KO). **b**, Tissue weights of control and *Ndufs2* KO mice at 4 months of age (*n* = 7 control and *n* = 5 *Ndufs2* KO). **c**, Representative images of H&E staining of gWAT and liver at 4 months of age (×10 magnification). **d**, Oral GTT and insulin during the GTT at 4 months of age (*n* = 7 control and *n* = 5 *Ndufs2* KO). **e**, Serum non-esterified fatty acid (NEFA) in control and *Ndufs2* KO mice at 4 months of age (*n* = 7 control and *n* = 5 *Ndufs2* KO). **f**, KEGG pathway analysis of downregulated (top) and upregulated (bottom) genes in *Ndufs2* KO versus control adipocytes (*n* = 4). **g**, RNA-seq was performed on fractionated adipocytes from the gWAT of control (*Ndufs2*^*loxP/loxP*^), *Ndufs2* KO (*Adipoq-cre;Ndufs2*^*loxP/loxP*^) and *Bmal1* KO (*Adipoq-cre; Bmal1*^*loxP/loxP*^) mice. Pathway analysis of Gene Ontology biological process terms with highlighted genes among differentially expressed genes in *Ndufs2* KO versus control and *Bmal1* KO versus control fractionated adipocytes. **h**, Motif analysis of downregulated genes in *Ndufs2* KO versus control and *Bmal1* KO versus control fractionated adipocytes. Data are presented as the mean ± s.e.m. **a**,**e**, Statistical significance was calculated using an unpaired two-sided *t*-test. **b**,**d**, A two-way ANOVA followed by multiple comparisons test was used. **h**, A two-sided hypergeometric test with Benjamini–Hochberg correction for multiple testing was used. *P < 0.05, **P < 0.01, ***P < 0.001. Panel **g** includes elements created in BioRender; M, B. https://BioRender.com/s27y548 (2025). Source data

**Extended Data Fig. 7:**
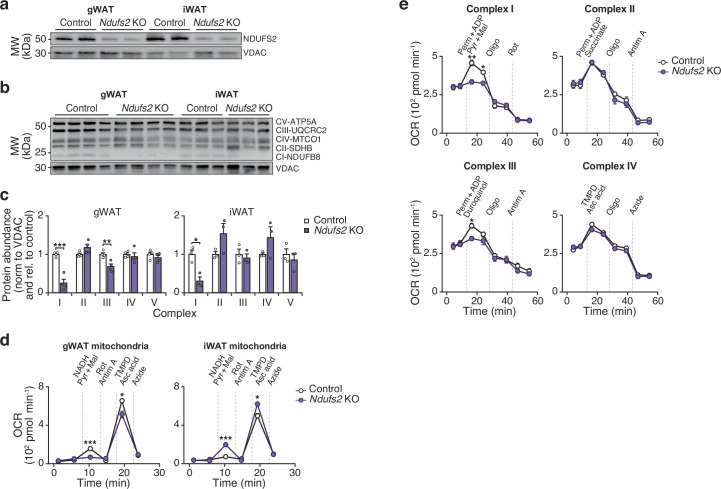
Loss of *Ndufs2* in adipocytes leads to reduced complex I and III activity in gWAT, related to Fig. 3. **a**, SDS-PAGE immunoblot of *Ndufs2* in isolated mitochondria from Control (*Ndufs2*^*fx/fx*^) and *Ndufs2*-KO (*Adiponectin-Cre; Ndufs2*^*fx/fx*^) mice at 3 months of age on a chow diet (n = 2). **b**, SDS-PAGE immunoblot of electron transport chain proteins in isolated gWAT mitochondria from Control and *Ndufs2*-KO mice at 3 months of age (n = 3-4). **c**, Quantification of protein abundance of samples in B. **d**, OCR in gWAT and iWAT mitochondria of Control and *Ndufs2*-KO mice at ZT14 in the presence of indicated substrates and inhibitors (n = 7 for gWAT and n = 6 for iWAT). TMPD, Tetramethyl-p-phenylenediamine; Asc acid; ascorbic acid. **e**, OCR of differentiated adipocytes from Control and *Ndufs2*-KO mice in the presence of permeabilizer (PERM), ADP, and complex I to IV substrates followed sequentially by oligomycin and antimycin A (n = 5). Data are represented as mean ± SEM. Statistical significance was calculated by two-way ANOVA followed by multiple comparisons in **c**, **d**, and **e**.*p < 0.05, **p < 0.01, ***p < 0.001. Source data

*Ndufs2* deletion led to loss of NDUFS2 protein in isolated adipose tissue mitochondria, along with reduced CI subunit NDUFB8 and CIII subunit UQCRC2 (Extended Data Fig. 7a–c). Consistent with this finding, we observed less respiration in response to CI and III substrates in isolated gWAT mitochondria and primary adipocytes from mice lacking adipocyte *Ndufs2* (Extended Data Fig. 7d,e). Mice lacking adipocyte *Ndufs2* exhibited similar body weight to control mice on a chow diet (Fig. 3a). However, adipocyte *Ndufs2* KO mice had reduced WAT mass, increased liver mass, visceral adipocyte hypertrophy and impaired glucose tolerance (Fig. 3b–d). In addition, circulating fatty acids were elevated in *Ndufs2* KO mice (Fig. 3e). These findings indicate that loss of CI in adipocytes includes lipid storage capacity in adipose tissue and systemic metabolic homeostasis.

To define the molecular basis of the metabolic dysfunction caused by CI loss in adipocytes, we next analysed the transcriptome in visceral WAT. We performed RNA sequencing (RNA-seq) on the fractionated adipocytes from the gWAT of these mice. In adipocytes lacking CI, we observed reduced expression of genes involved in the response to insulin stimulus and PI3K/protein kinase B signal transduction (Fig. 3f and Supplementary Table 1). Expression of *Akt1/2*, *Irs1/2* and *Insr*, as well as several transcription factors important in adipocyte metabolism and function such as *Pparg*, *Foxo1*, *Cebpb* and *Tcf7l2* were reduced in adipose *Ndufs2* KO mice. In addition, there was upregulation in the expression of the proinflammatory cytokine *Tnf* and genes involved in the integrated stress response (*Atf3*, *Atf5*, *Ddit3* and *Dele1*) and reduced expression of the adipokine *Adipoq*. This transcriptional profile during CI dysfunction suggests a broader impairment in adipocyte metabolism and function, extending beyond isolated electron transport chain (ETC) disruption.

**Extended Data Fig. 8:**
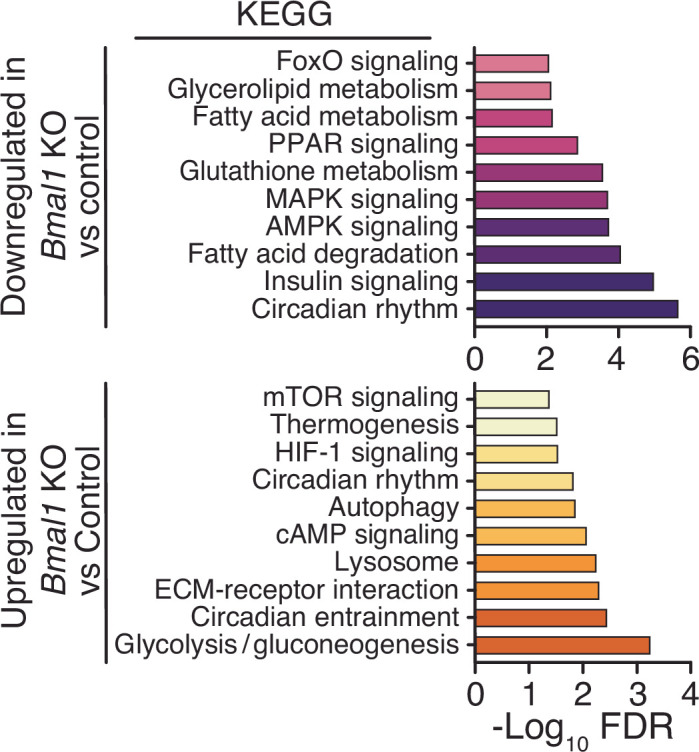
*Bmal1* loss in adipocytes remodels circadian, metabolic. and stress signaling pathways, related to Fig. 3. KEGG pathway analysis of downregulated (upper panel) and upregulated (lower panel) genes in *Bmal1*-KO vs. Control adipocytes (n = 4).

Deletion of *Ndufs2* caused a complete loss of CI activity with secondary reduction of CIII in gWAT. This resulted in reduced WAT mass, increased liver mass, impaired glucose tolerance and elevated circulating FAs, which is consistent with a primary defect in adipose lipid storage. In contrast, deletion of *Bmal1* led to only a partial reduction in CI respiration and a milder metabolic phenotype. These physiological differences were reflected in the transcriptome. *Ndufs2* KO adipocytes showed reduced expression of the pathways involved in fatty acid metabolism, insulin signalling, peroxisome proliferator-activated receptor (PPAR) signalling and lipolysis, along with activation of oxidative stress, inflammatory and glycolytic programmes. *Bmal1* KO adipocytes display a distinct pattern via downregulation of circadian rhythm, insulin signalling, glycerolipid metabolism and glutathione metabolism, with upregulation of thermogenesis, autophagy and cAMP signalling (Extended Data Fig. 8 and Supplementary Table 1).

Both genetic deletion of CI and *Bmal1* resulted in downregulation of genes involved in the insulin response and mRNA transcription, and an increase in genes involved in translation (Fig. 3g and Supplementary Table 1). Among the overlapping downregulated genes were *Pparg* and *Cebpa*, master regulators of fat cell metabolism, insulin sensitivity and adipogenesis, and *Ppargc1b*, a transcriptional coactivator for PPARs^31^. We also noted an increase in the integrated stress response genes *Atf5* and *Dele1* and several CI subunits. Motif analysis of downregulated genes in both *Ndufs2* and *Bmal1* KO adipocytes revealed shared enrichment for C/EBP and SP family transcription factor motifs (Fig. 3h). These factors are key regulators of *Pparg* expression and adipogenic gene networks, which is consistent with the observed transcriptional changes. The shared depletion of C/EBP and SP motifs suggests that disruption of CI and circadian clock function converge to maintain adipogenic transcriptional identity and insulin-responsive gene expression.

**Fig. 4:**
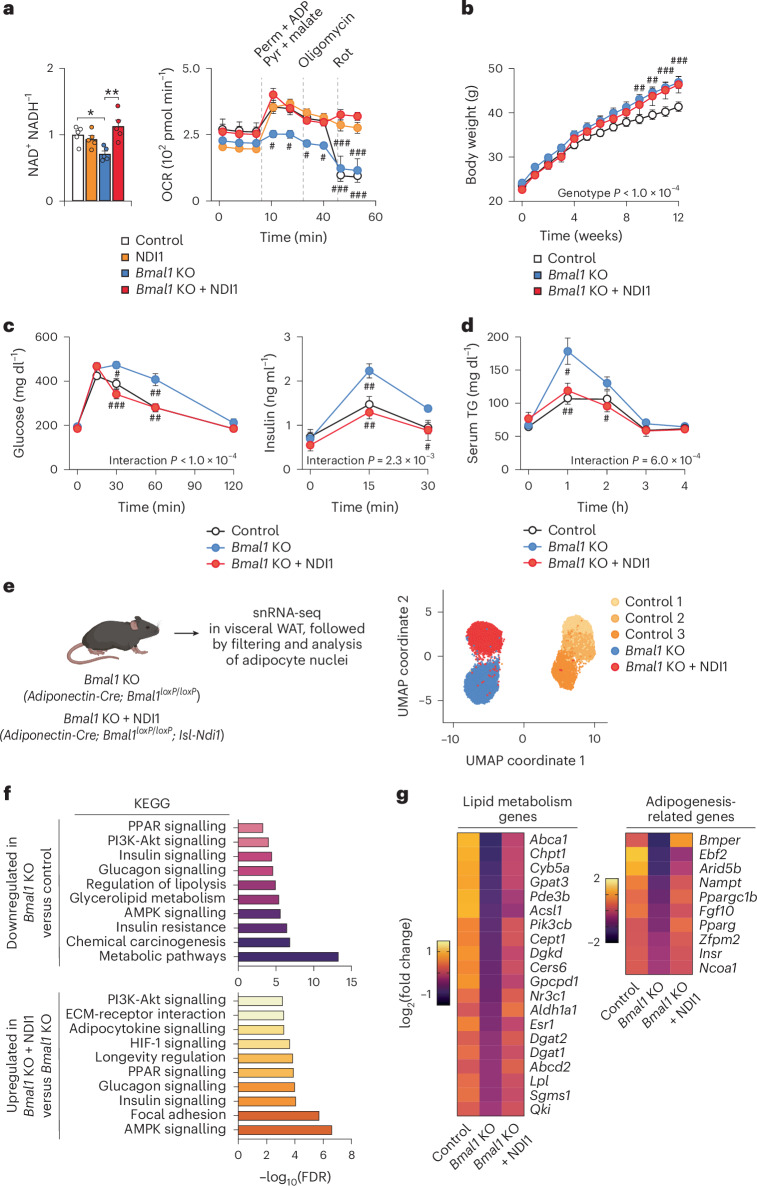
Expression of NDI1 prevents diet-induced metabolic dysfunction in mice lacking the circadian clock in adipocytes. **a**, Intracellular NAD^+^-to-NADH ratio and OCR in the presence of the indicated substrates and inhibitors in primary white adipocytes differentiated from control, NDI1, *Bmal1* KO and *Bmal1* KO + NDI1 mice (*n* = 5). **b**, Body weight during HFD feeding for 12 weeks (control *n* = 12, *Bmal1* KO *n* = 12, *Bmal1* KO + NDI1 *n* = 7). **c**, Oral GTT and insulin during the GTT at 6 weeks of HFD feeding (control *n* = 12, *Bmal1* KO *n* = 12, *Bmal1* KO + NDI1 *n* = 7). **d**, Oral triglyceride (TG) clearance test at 6 weeks of HFD feeding (control *n* = 12, *Bmal1* KO *n* = 12, *Bmal1* KO + NDI1 *n* = 7). **e**, snRNA-seq was performed on gWAT from control (*Bmal1*^*loxP/loxP*^*; NDI1*^*LSL*^), *Bmal1* KO (*Adipoq-cre; Bmal1*^*loxP/loxP*^) and *Bmal1* KO + NDI1 (*Adipoq-cre; Bmal1*^*loxP/loxP*^*; NDI1*^*LSL*^) mice. Uniform manifold approximation and projection (UMAP) plot showing WAT adipocyte nuclei from control, *Bmal1* KO and *Bmal1* KO + NDI1 mice (the WAT from four mice was pooled for each sample). **f**, KEGG pathway analysis of downregulated genes in *Bmal1* KO versus control adipocytes (top) and upregulated genes in *Bmal1* KO + NDI1 versus *Bmal1* KO adipocytes (bottom). **g**, Heatmap of differentially abundant genes (adjusted *P* < 0.05) in adipocyte clusters between control, *Bmal1* KO and *Bmal1* KO + NDI1 mice. Data are presented as the mean ± s.e.m. **a**–**d**, Statistical significance was calculated using a one-way ANOVA followed by multiple comparisons in **a** (left) and a two-way ANOVA followed by Dunnett’s multiple comparisons test with the *Bmal1* KO set as the reference group in **a** (right) and **b**–**d**. The # symbol denotes statistical significance between *Bmal1* KO and the indicated groups. *P < 0.05, **P < 0.01, ***P < 0.001. Panel **e** includes elements created in BioRender; M, B. (2025) https://BioRender.com/s27y548 (2025). Source data

**Extended Data Fig. 9:**
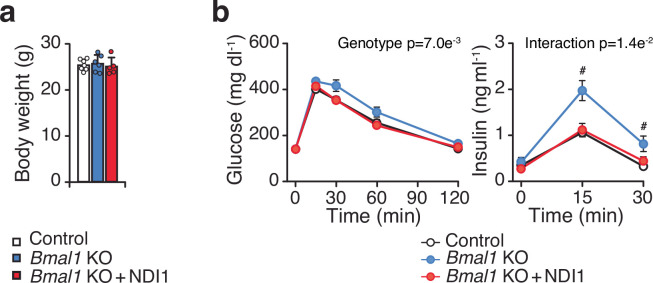
Deletion of Bmal1 leads to impaired glucose tolerance on a chow diet, which is prevented by NDI1, related to Fig. 4. **a**, Body weight of Control, *Bmal1*-KO, and *Bmal1*-KO + NDI1 mice at 4 months of age on a chow diet (n = 7 Control, n = 6 *Bmal1*-KO, and n = 5 *Bmal1*-KO + NDI1). **b**, Glucose tolerance test (GTT) and insulin during the GTT of the mice in A at 4 months of age (n = 7 Control, n = 6 *Bmal1*-KO, and n = 5 *Bmal1*-KO + NDI1). Data are represented as mean ± SEM. Statistical significance was calculated by one-way ANOVA in **a** and two-way ANOVA followed by Dunnett’s multiple comparisons test with Bmal1-KO mice set as the reference group in **b**. # denotes statistical significance between Bmal1-KO vs other groups (p < 0.05). Source data

Prior work has shown that mice lacking the clock in adipocytes exhibit impaired glucose homeostasis during chow and HFD feeding^8,11^. To test whether a defect at CI underlies this phenotype, we generated mice deficient in adipocyte *Bmal1* with NDI1-expressing mice. NDI1 expression in *Bmal1* KO adipocytes restored the NAD^+^-to-NADH ratio and CI respiration (Fig. 4a). On a chow diet, adipocyte-specific loss of *Bmal1* impaired glucose tolerance and caused compensatory hyperinsulinaemia, which was normalized by NDI1 (Extended Data Fig. 9a,b). Therefore, NDI1 rescues the metabolic defects caused by clock disruption independently of dietary stress.

**Extended Data Fig. 10:**
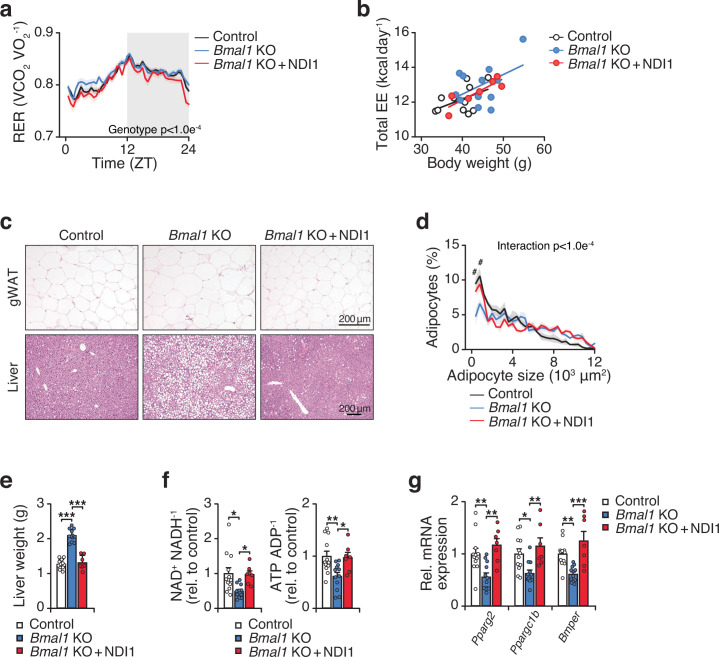
Preserving complex I activity with NDI1 mitigates metabolic and adipose dysfunction in Bmal1-KO mice, related to Fig. 4. **a**, RER over 24 hours after 10 weeks of HFD feeding (Control n = 12, *Bmal1*-KO n = 12, *Bmal1*-KO + NDI1 n = 7). **b**, Total energy expenditure over 24 hours versus body weight after 10 weeks of HFD feeding (Control n = 12, Bmal1-KO n = 12, Bmal1-KO + NDI1 n = 7). **c**, Representative images of hematoxylin and eosin staining of gWAT and liver after 12 weeks of HFD feeding (10x magnification, scale bars 200 μm). **d**, Distribution in gWAT adipocyte size after 12 weeks of HFD feeding (n = 5). **e**, Liver weights after 12 weeks of HFD feeding (Control n = 12, *Bmal1*-KO n = 12, *Bmal1*-KO + NDI1 n = 7). **f**, The NAD + /NADH and ATP/ADP ratios in WAT after 12 weeks of HFD feeding at ZT14 (Control n = 12, *Bmal1*-KO n = 12, *Bmal1*-KO + NDI1 n = 7). **g**, qPCR analysis of indicated genes in gWAT from Control, *Bmal1*-KO, and *Bmal1*-KO + NDI1 mice (Control n = 12, *Bmal1*-KO n = 12, *Bmal1*-KO + NDI1 n = 7). Data are represented as mean ± SEM. Statistical significance was calculated by two-way ANOVA followed by Dunnett’s multiple comparisons test with *Bmal1*-KO set as the reference group in **a** (right panel), and **a** and **d**, and ANCOVA in **b**, and one-way ANOVA followed by multiple comparisons in **e-g**. The # symbol denotes statistical significance between *Bmal1*-KO and indicated groups. *p < 0.05, **p < 0.01, ***p < 0.001. Source data

During HFD feeding, mice lacking the circadian clock in adipocytes gained excess body weight and had impaired glucose homeostasis, which is consistent with prior results^8,11^ (Fig. 4b–d). Expression of NDI1 in adipocyte-*Bmal1* KO mice had no effect on weight gain or energy expenditure during HFD feeding compared with adipocyte-*Bmal1* KO mice (Fig. 4b and Extended Data Fig. 10a,b). However, NDI1 expression normalized glucose tolerance, insulin release and lipid clearance comparable to that of control mice (Fig. 4c,d). In NDI1-expressing adipocyte-*Bmal1* KO mice, we also observed reduced hepatic steatosis, smaller white adipocyte size and restored ratios of NAD^+^-to-NADH and ATP-to-ADP (Extended Data Fig. 10c–f). Collectively, these findings reveal that circadian disruption in adipocytes promotes metabolic syndrome through disruption of CI function.

To further assess how CI affects transcription, we analysed RNA expression in circadian mutant mice with restored CI function. We performed single-nucleus RNA-seq (snRNA-seq) in gWAT from chow-fed control, adipocyte-*Bmal1* KO and adipocyte-*Bmal1* KO + NDI1 mice. After doublet removal and filtering of the data, we obtained a total of 12,995 adipocyte nuclei (5,874 control, 3,949 *Bmal1* KO and 3,172 *Bmal1* KO + NDI1) that grouped into five distinct clusters (Fig. 4e and Supplementary Table 2). Dominant clustering was driven by genotype; however, additional clusters may have been revealed with a greater number of nuclei or with inclusion of other depots, diets or experimental conditions.

Among the adipocyte nuclei in control WAT, we observed three subpopulations with diverse gene expression profiles (Supplementary Table 3). Kyoto Encyclopedia of Genes and Genomes (KEGG) pathway analysis revealed that cluster 1 (1,747 nuclei) expressed genes involved in fatty acid metabolism, phosphatidylinositol-3-kinase (PI3K)-AKT signalling and lipolysis. Cluster 2 (2,203 nuclei) expressed genes involved in PPAR signalling, insulin signalling and glycerolipid metabolism. Cluster 3 (1,913 nuclei) was characterized by genes involved in oxidative phosphorylation and reactive oxygen species. Next, we compared gene expression between control and *Bmal1* KO adipocytes. Adipocytes lacking *Bmal1* had reduced expression of genes involved in fatty acid metabolism, lipid biosynthesis and fat cell differentiation (Fig. 4f). In addition, pathways involved in PPAR, insulin and glucagon signalling were downregulated in *Bmal1* KO adipocytes. Notably, *Bmal1* KO adipocytes had reduced expression of *Pparg*, *Tcf712* (a regulator of lipid and glucose metabolism^32^) and several genes involved in lipid metabolism and adipogenesis (Fig. 4g and Supplementary Table 4).

Strikingly, pathways involved in metabolism, fat cell differentiation, insulin signalling and PPAR signalling that were downregulated in *Bmal1* KO adipocytes were restored with NDI1 expression (Fig. 4f). NDI1 expression increased the levels of several adipogenic and metabolic genes, including *Pparg*, *Bmper* (a positive modulator of adipogenesis^33^), *Ppargc1b* and enzymes involved in lipid metabolism (*Dgat1*, *Dgat2* and *Lpl*) (Fig. 4g). Quantitative PCR (qPCR) analysis of fractionated adipocytes in lean mice confirmed that NDI1 expression prevents the decline in *Pparg*, *Ppargc1b* and *Bmper* in mice lacking adipocyte *Bmal1* (Extended Data Fig. 10g). Therefore, restoration of CI function bypasses defects in lipogenic and nutrient-responsive transcription networks caused by circadian disruption in adipocytes.

## Discussion

We found that the adipocyte clock programmes oxidative metabolism through rhythmic respiration at mitochondrial CI. Dysfunction of CI is a defining feature of circadian disruption and diet-induced obesity in adipose tissue, linking clock misalignment to visceral adipocyte hypertrophy, ectopic lipid deposition in liver and impaired glucose tolerance. Preserving NADH entry into the respiratory chain with NDI1 prevented these manifestations despite similar weight gain, indicating that mitochondrial electron entry controls metabolic health independently of body weight.

Defects in CI biogenesis are the most common cause of mitochondrial disorders and are linked to age-related diseases, including neurodegeneration^34^. Mechanistically, circadian control of CI probably reflects the intersection of substrate routing and time-of-day coordination of CI biogenesis, stability and activity. CI is a large 45-subunit L-shaped complex in the mitochondrial inner membrane^15^. It serves as the main gateway for electron entry into the ETC and is the primary site for regeneration of NAD^+^. Previous work has shown that the clock regulates NAD^+^ biosynthesis and deacetylation pathways that, in turn, regulate mitochondrial enzyme activity^4,35^. This NAD^+^ regulation is critical for clock-dependent acetylation, a modification that may influence the assembly and activity of CI^36^. Further work is needed to define the molecular mechanisms through which the clock coordinates CI abundance and function.

In adipose tissue, we observed that CI respiration was rhythmic across gWAT and iWAT, and was reduced by *Bmal1* loss, whereas respiration through CII–CIV was not affected by loss of the clock. The total abundance of CI showed phase-dependent and clock-dependent differences, consistent with temporal regulation of assembly or stability rather than transcriptional control. We observed reduced total CI abundance, reflected by a decrease in NDUFA9 with parallel trends in additional subunits. This broader reduction is consistent with impaired CI assembly or stability, rather than an isolated subunit defect. Because CI is the largest and most energetically costly respiratory complex to assemble, coordinating its maintenance with phases of high energy and cofactor availability may provide an efficient mechanism to sustain rhythmic respiration. In other tissues, including the liver, skeletal muscle and macrophages, genetic disruption of clock genes has also been linked to changes in CI, CII and CIII respiration^6,37–43^, highlighting that clock regulation of mitochondrial function is shaped by cell type and metabolic state. In adipocytes, preferential routing of glucose towards lactate production and citrate export for lipogenesis limits succinate flux through CII, which may bias electron entry towards CI^44,45^. We speculate that this reliance on CI-dependent NAD⁺ regeneration, together with the dynamic demands of lipid synthesis and storage, makes CI susceptible to circadian regulation in adipose tissue.

We found that visceral adipose tissue is particularly sensitive to changes in CI function. In humans, metabolically healthy obesity is associated with smaller adipocytes and a lower visceral-to-subcutaneous fat ratio, whereas expansion of visceral fat correlates with systemic insulin resistance and ectopic lipid deposition^46–48^. In male mice, gWAT undergoes the greatest expansion during HFD feeding and supports the bulk of lipogenesis during the active/feeding phase, placing a high demand on continuous NAD^+^-dependent tricarboxylic acid cycle (TCA) flux^21,49–51^. Such lipogenesis increases energy demand which results in increased demand for NAD^+^ in the TCA cycle. Therefore, reduced CI-driven NADH oxidation would constrain lipogenic capacity most prominently in this depot, which is consistent with our observations that impairment in CI reduces adipogenesis and promotes hypertrophic remodelling in gWAT. Mitochondrial proteomic datasets reveal that adipose depots have distinct respiratory chain compositions and CI regulatory inputs, further supporting the idea that reduced CI function differentially affects distinct adipose tissue depots^52,53^. We found that CI loss also reduced CIII-linked respiration in gWAT but not in iWAT, indicating that downstream respiratory capacity is disproportionately affected in visceral fat.

Mitochondria have emerged as dynamic signalling organelles that regulate cellular function beyond their metabolic roles^54^. Loss of CI resulted in reduced expression of transcription networks involved in insulin sensitivity, PPAR signalling and fatty acid metabolism. Both genetic circadian disruption and HFD feeding shifted adipocyte cell state from a pro-adipogenic signature towards an insulin resistant state^55,56^. Preserving CI function with NDI1 expression maintained adipogenic and lipogenic transcription in adipocytes lacking the circadian clock. Notably, NDI1 maintains CI respiratory capacity independently of time of day, even when rhythmicity is blunted by HFD or *Bmal1* loss. NDI1 expression also supported healthier adipose remodelling during HFD feeding, reflected by a shift towards smaller adipocytes with no difference in gWAT mass. NDI1 also normalized the metabolic defects caused by adipocyte *Bmal1* deletion on chow diet and improved metabolic health during HFD feeding. These findings indicate that NDI1 mitigates metabolic dysfunction driven by both circadian disruption and HFD, although it does not fully counteract the broader metabolic load of obesity. All of our experiments were performed in male mice; given known sex differences in metabolic and circadian physiology, whether female mice exhibit similar regulation of CI in adipose tissue is to be determined.

NDI1 expression led to increased NAD^+^, nucleotides and α-ketoglutarate with reduced succinate. These metabolite changes suggest that the retrograde signal may involve altered activity of chromatin-modifying enzymes sensitive to these metabolites. Defining the mitochondrial signals linking CI respiration to transcription will be key to understanding how circadian clocks coordinate cellular identity with metabolic state.

Collectively, our studies identify an essential function of the circadian clock in mitochondrial respiration through CI. These findings have implications for understanding how mitochondrial metabolism within adipose tissue feeds back to regulate epigenetic state.

## Methods

### Animals and diets

All animal experiments were performed according to procedures approved by the Northwestern University Institutional Animal Care and Use Committee, using male mice on a C57BL/6J background. *Adipoq-cre* mice (strain no. 028020, The Jackson Laboratories) were bred to *Bmal1*^*loxP*/*loxP*^ (C. Bradfield, University of Wisconsin), *NDI1*^*LSL*^ (N. Chandel, Northwestern University)^24^ and *Ndufs2*^*loxP/loxP*^ (J. López-Barneo, Universidad de Sevilla) mice. mPer2^Luc^ (strain no. 006852, The Jackson Laboratories) mice were used for in vitro circadian synchronization experiments. Mice were group-housed and maintained at room temperature (23–25 °C) on a standard chow diet with a 12-h light–dark cycle and free access to water and food unless specified. In the HFD feeding experiments, mice were fed HFD (45% kcal from fat, cat. no. F6635, Bio-Serv) beginning at 3 months of age. No statistical methods were used to predetermine sample sizes but our sample sizes are similar to those reported in our previous publications^8,57^. Researchers were not blinded to animal groups. Unless otherwise specified, data collection and analysis were not performed blind to the conditions of the experiments.

### Indirect calorimetry

Mice were housed singly in Sable Systems metabolic cages at room temperature (24 °C). Mice were acclimated to the metabolic cages for 5 days and were provided ad libitum access to food and water. Food intake, VO_2_, VCO_2_, RER (VCO_2_/VO_2_) and locomotor activity (beam breaks) were continuously monitored throughout the experiment.

### Metabolic phenotyping

For the glucose tolerance tests, mice were fasted for 2 h (beginning at ZT0) and then administered glucose via oral gavage (2.5 g kg^−1^ body weight). At the indicated time points, tail blood was collected. Blood glucose was measured using a Bayer Contour glucometer. Serum insulin was measured using an Ultra-Sensitive Mouse Insulin ELISA (CristalChem). For the TG clearance tests, mice were fasted overnight (~15 h), then administered 20% intralipid (Sigma-Aldrich) via oral gavage (15 µl g^−1^ body weight) at ZT2. Blood was collected hourly and then assayed for serum TGs (Infinity Triglycerides Reagent, Thermo Fisher Scientific).

### Cold tolerance testing

Mice were implanted subcutaneously in the dorsal region with IPTT-300 temperature transponders (BMDS). Body temperature was recorded using a DAS-7006/7s reader (BMDS). Mice were then transferred to a 6 °C cold chamber at 10 weeks of age. For the acute cold tolerance test, food was removed at the time of transfer and body temperature was measured at the indicated time points. For the cold-acclimated exposure test, mice were maintained at 6 °C for 2 weeks before testing. Food was removed at the start of the test and body temperature was monitored at the indicated time points.

### Histological analysis

Adipose tissues and liver were collected and placed in 4% paraformaldehyde overnight, followed by 70% ethanol for up to 4 days before processing. Paraffin embedding, tissue sectioning and H&E staining were performed by the Mouse Histology and Phenotyping Laboratory core at Northwestern University. Images were obtained on a Keyence BZ-X810. Adipocyte size analysis was performed using the Keyence BZ-X Analyzer software based on brightfield images of H&E-stained paraffin sections. Quantification was performed by an investigator blinded to sample identity.

### Gene expression analysis by qPCR

RNA was isolated from frozen tissues or fractionated adipocytes using TRIzol and the RNeasy Mini Kit (QIAGEN). To isolate fractionated adipocytes, fresh gWAT was minced and digested for 1 h in PBS containing 10 mM CaCl_2_, 2.4 U ml^−1^ Dispase II (Roche) and 1.5 U ml^−1^ collagenase D (Roche). The homogenate was filtered through a 100-μm cell strainer and centrifuged at 500*g*, followed by transfer of the floated adipocytes to an Eppendorf tube for RNA isolation. cDNA was synthesized using M-MLV Reverse Transcriptase (Invitrogen) and Random Primers (Invitrogen). Relative mRNA levels were determined using qPCR with the iTaq Universal SYBR Green Supermix (Bio-Rad Laboratories). Values were normalized to three housekeeping genes (*Actb*, *Rplp0* and *Ppia*) using the ΔΔ*C*_t_ method. All primer sequences were designed using Primer3 (ref. ^58^) primer design software and are listed in Supplementary Table 5.

### WAT mitochondria isolation

To isolate mitochondria, fresh gWAT was minced then homogenized using a drill-operated Teflon pestle in ice-cold MSHE buffer (70 mM sucrose, 210 mM mannitol, 5 mM HEPES, 1 mM EDTA, pH 7.2) containing 0.5% FA-free BSA. The homogenate was centrifuged at 800*g* for 10 min, followed by centrifugation of the supernatant at 8,000*g* for 10 min. The mitochondrial pellet was resuspended in MSHE buffer with BSA and protein concentration was determined using the Pierce BCA Protein Assay Kit (Thermo Fisher Scientific).

### Mitochondrial function and respiration

Isolated WAT mitochondria, cultured primary differentiated adipocytes and MEFs were assayed for OCR using a Seahorse XFe96 Extracellular Flux Analyzer (Agilent Technologies). For the mitochondrial OCR assays, mitochondria were plated on Seahorse Biosciences 96-well culture plates (5 μg of protein per well) and centrifuged for 20 min at 2,000*g* at 4 °C to adhere mitochondria to the base of wells. For the electron coupling assays, respiratory substrates (10 mM pyruvate + 2 mM malate, 10 mM succinate + 2 μM rotenone, 80 μM palmitoyl-carnitine or octanoyl-carnitine + 0.5 mM malate or 10 mM glutamate + 10 mM malate) were diluted in MAS buffer with BSA (70 mM sucrose, 220 mM mannitol, 10 mM KH_2_PO_4_, 5 mM MgCl_2_, 2 mM HEPES and 1 mM EGTA, pH 7.2, in 0.2% FA-free BSA) and added to mitochondria. Substrate injection was as follows: 4 mM ADP at port A, 10 μM oligomycin at port B, 10 μM FCCP at port C and 10 μM antimycin A at port D. To determine CI activity, MAS plus alamethicin (2.5 μg ml^−1^) and cytochrome *c* (10 μg ml^−1^) were added to the mitochondria followed by 5 mM pyruvate + 5 mM malate + 1 mM NADH at port A, 2 μM rotenone + 4 μM antimycin A at port B, 0.5 mM TMPD + 1 mM ascorbic acid at port C and 50 mM azide at port D. For the electron flow assays, mitochondria were plated in MAS buffer with 10 mM pyruvate + 2 mM malate + 4 μM FCCP followed by 20 μM rotenone in port A, 100 mM succinate in port B, 40 μM antimycin A in port C and 1 mM TMPD + 100 mM ascorbic acid in port D.

For the OCR assays in cells, culture medium was replaced with MAS buffer with 4% BSA, pH 7.2. Activity measurements for respiratory chain CI–IV in permeabilized cells were performed as described previously^59^. The first injection in port A contained the substrate for each individual respiratory chain complex (I: 5 mM pyruvate + 2.5 mM malate, II: 10 mM succinate + 1 μM rotenone, III: 0.5 mM duroquinol, IV: 0.5 mM TMPD + 2 mM ascorbic acid) along with 1 mM ADP and 10 nM recombinant perfringolysin O (Agilent Technologies). To determine CI activity (NADH-dependent OCR), MAS plus alamethicin (2.5 μg ml^−1^) and cytochrome *c* (10 μg ml^−1^) was added onto the cells followed by 5 mM pyruvate + 5 mM malate + 1 mM NADH + 10 nM recombinant perfringolysin O at port A, 2 μM rotenone + 4 μM antimycin A at port B, 0.5 mM TMPD + 1 mM ascorbic acid at port C and 50 mM azide at port D. The OCR of MEFs was normalized to total protein content because of differences in proliferation.

### Immunoblotting

Protein extracts from adipose tissue mitochondria or cells were prepared using homogenization in radioimmunoprecipitation assay buffer containing protease inhibitors (Sigma-Aldrich). The homogenate was spun at 18,000*g* for 15 min and the supernatant was used for subsequent analysis. Protein levels were quantified using a bicinchoninic acid assay. Immunoprecipitation of NDUFS2 was performed using the Pierce IP/co-IP kit (Thermo Fisher Scientific) according to the manufacturer’s instructions, with an anti-NDUFS2 antibody (1:40 dilution, cat. no. ab192022, Abcam). Protein extracts were then subjected to SDS–PAGE and transferred to Immobilin-FL polyvinylidene fluoride transfer membrane. The immunoblots were incubated with primary antibody at 4 °C overnight followed by incubation with IR Dye-coupled secondary antibody and visualization using the LI-COR Odyssey Fc imaging system. The following antibodies were used: OXPHOS cocktail (1:250 dilution, cat. no. ab110413, Abcam), NDUFA9 (1:1,000 dilution, cat. no. ab14713, Abcam), NDUFA10 (1:1,000 dilution, cat. no. sc-376357, Santa Cruz Biotechnology), NDUFS2 (1:1,000 dilution, cat. no. ab192022, Abcam), UQCRC1 (1:1,000 dilution, cat. no. ab110252, Abcam), VDAC (1:1,000 dilution, cat. no. PA1-954A, Thermo Fisher Scientific), mono and dimethyl arginine (1:500 dilution, cat. no. ab412, Abcam), goat anti-mouse IRDye 680RD (1:10,000 dilution, cat. no. 926-68070, LI-COR) and goat anti-rabbit IRDye 800CW (1:10,000 dilution, cat. no. 926-32211, LI-COR).

### BN-PAGE

For BN-PAGE, isolated mitochondria were resuspended in buffer (20 mM Tris, pH 7.4; 0.1 mM EDTA; 50 mM NaCl; 10% (v/v) glycerol) containing 2% *N*-dodecyl β-d-maltoside for a 20-min incubation on ice, followed by centrifugation at 20,000*g* for 10 min. The protein concentration of the supernatant was measured using a bicinchoninic acid protein assay. Then, 15 μg of protein was mixed with NativePAGE sample buffer (Invitrogen), Coomassie G-250 and protease inhibitor, loaded into the lanes of a 3–12% Bis-Tris NativePAGE gel (Invitrogen) and run according to the manufacturer’s instructions. OXPHOS complexes were detected using the Blue Native OXPHOS antibody cocktail (1:250 dilution, cat. no. ab110412, Abcam). Proteins were visualized using immunoblot analysis.

### MEF cell culture

Control, *Bmal1*^−/−^ and *Cry1*^−/−^*Cry2*^−/−^ MEFs were cultured in DMEM (Sigma-Aldrich) supplemented with 15% FCS (Atlanta Biological) and 1% penicillin-streptomycin at 37 °C with 5% CO_2_. Culture medium was exchanged every 1–2 days. All cells used in these experiments were less than 15 passages.

### Isolation of WAT stromal vascular cells and adipocyte differentiation

To isolate the stromal vascular fraction, minced iWAT (combined depots from 4–5 mice per genotype) from 4–6-week-old mice was digested in 1 mg ml^−1^ collagenase D (Roche) with 1.5% BSA in sterile Hank’s Balanced Salt Solution and incubated in a 37 °C shaking water bath for 1 h. The mixture was then passed through a 100-µm cell strainer, pelleted at 500*g*, resuspended in PBS, passed through a 40-µm cell strainer and again pelleted at 500*g*. Stromal vascular fraction cells were expanded in growth medium containing DMEM/F12 with GlutaMAX (Thermo Fisher Scientific) and 10% FCS with 100 U ml^−1^ penicillin-streptomycin and 50 μg ml^−1^ gentamicin, and incubated in 10% CO_2_ at 37 °C. Upon reaching confluence, cultures were incubated with the adipogenesis induction cocktail (growth medium supplemented with 5 μg ml^−1^ insulin, 1 μM dexamethasone, 0.5 mM isobutylmethyxanthine and 1 μM rosiglitazone) for day 1–2 of differentiation. For days 2–4 of differentiation, cells were incubated with 5 µg ml^−1^ insulin and 1 µM rosiglitazone. For days 4–8 of differentiation, cells were maintained in growth medium supplemented with 5 µg ml^−1^ insulin until collection. Adipocytes were considered mature for use in assays on day 8 of differentiation. For the synchronization experiments, cells were synchronized with dexamethasone (100 nM, 30 min) on day 6 of differentiation and assayed or collected at the indicated time points. In parallel, Per2-luciferase rhythms were monitored in synchronized cells cultured in DMEM containing 0.1 mM luciferin using a LumiCycle apparatus (Actimetrics).

### Isolation of polar metabolites

For the metabolomics of tissues, 100–200 mg of weighed tissue was homogenized in 1 ml of ice-cold 80% methanol with a QIAGEN Tissue Lyser II (three sets of 2 min on maximum speed, with 2 min of rest on dry ice in between sets). The homogenized tissue was then incubated at −80 °C for 15 min and then centrifuged at 18,000*g* at 4 °C for 5 min. The lipid layer was avoided and the supernatant was transferred to a new tube on dry ice and then centrifuged at 18,000*g* at 4 °C for 5 min. The equivalent volume of 20 mg of tissue was transferred to a new tube, which was then dried in a SpeedVac.

For the metabolomics of cell cultures, cells were differentiated in 6-well plates. On day 6 of differentiation, the medium was changed to glucose/phenol red/glutamine/pyruvate-free DMEM (Thermo Fisher Scientific) supplemented with 0.5% FCS, 4.5 g l^−1^ glucose, 4 mM glutamine, 0.5 mM pyruvate and 5 μg ml^−1^ insulin. After 48 h, the medium was removed, 400 µl of ice-cold 80% methanol was added and the cells were incubated at −80 °C for 15 min. Lysed cells were scraped on dry ice and transferred to Eppendorf tubes for centrifugation at 18,000*g* at 4 °C for 5 min. The entire supernatant was transferred to a new tube and dried in a SpeedVac. The pellet was resuspended in 200 µl of 8 M urea with 10 mM Tris (pH 8), incubated at 60° C for 30 min with shaking and centrifuged for 15 min at 18,000*g* for protein quantification of the supernatant with the DC Protein Assay (Bio-Rad Laboratories).

### Liquid chromatography–mass spectrometry analysis of metabolites

All liquid chromatography–mass spectrometry (LC–MS) sample analyses and metabolite quantification were performed with sample identities blinded to the analyst. The dried metabolites were reconstituted in 60% acetonitrile followed by overtaxing for 30 s and then centrifugation for 30 min at 20,000*g* at 4 °C. The supernatant was analysed using high-performance liquid chromatography and high-resolution mass spectrometry and tandem mass spectrometry. Specifically, the system consisted of a Thermo Q-Exactive in line with an electrospray source and an Ultimate3000 series HPLC consisting of a binary pump, degasser and auto-sampler outfitted with an Xbridge Amide column (Waters) (dimensions: 3.0 × 100 mm and a 3.5-µm particle size). The mobile phase A contained 95% (vol/vol) water, 5% (vol/vol) acetonitrile, 10 mM ammonium hydroxide and 10 mM ammonium acetate, pH 9.0; B was 100% acetonitrile. The gradient was as follows: 0 min, 15% A; 2.5 min, 30% A; 7 min, 43% A; 16 min, 62% A; 16.1–18 min, 75% A; 18–25 min, 15% A with a flow rate of 150 μl min^−1^. The capillary of the electrospray Ionization source was set to 275 °C, with sheath gas at 35 arbitrary units, auxiliary gas at five arbitrary units and the spray voltage at 4.0 kV. In positive/negative polarity switching mode, an *m*/*z* scan range from 60 to 900 was chosen and MS1 data were collected at a resolution of 70,000. The automatic gain control target was set at 1 × 10^6^ and the maximum injection time was 200 ms. The top five precursor ions were subsequently fragmented, in a data-dependent manner, using the higher energy collisional dissociation cell set to 30% normalized collision energy in MS2 at a resolution power of 17,500. Besides matching *m/z*, metabolites were identified by matching either retention time with analytical standards or the MS2 fragmentation pattern. Data acquisition and analysis were carried out using the Xcalibur v.4.1 and Tracefinder v.4.1 software, respectively. Metabolites were normalized to total ion count of each sample. The NAD^+^-to-NADH and ATP-to-ADP ratios were calculated from the peak area values of each metabolite within the same individual sample and compared between groups.

### RNA-seq from fractionated adipocytes, library preparation and analysis

RNA was isolated from fractionated adipocytes as described above. RNA quality was assessed using TapeStation; those with an RNA integrity number greater than eight were used for library preparation. Libraries were constructed from 250 ng of RNA using the NEBNext RNA Ultra II Directional Library Prep Kit (New England Biolabs). Average library size and concentration were determined using TapeStation and qPCR (NEBNext Library Quant kit, New England Biolabs), respectively, before pooling. Pooled libraries were sequenced on a NextSeq 2000 (Illumina) using 100-bp single-end sequencing. Libraries were sequenced to an average depth of ~20 M aligned reads for differential gene analyses. Sequences were aligned to the mm39 transcriptome with STAR using the --quantMode Transcriptome and GeneCounts options. Gene counts were obtained from mm39 annotations (Ensembl 112) using rsem-calculate-expression. Differential expression was carried out using DESeq2 with default parameters; pathway analysis was performed using pathfindR. Motif enrichment of differentially expressed genes was performed using HOMER (findMotifsGenome.pl).

### snRNA-seq and analysis

For isolation of nuclei from gWAT, fresh adipose tissue was minced in nucleus preparation buffer (10 mM HEPES, 1.5 mM MgCl_2_, 10 mM KCl, 250 mM sucrose, 0.001% NP-40) with 0.2 U µl Roche Protector RNase inhibitor followed by dounce homogenization. The homogenate was filtered through a 100-μM cell strainer and centrifuged at 1,000*g* for 10 min at 4 °C, then the nuclear pellet was washed once in nucleus preparation buffer followed by resuspension in PBS with 1% BSA and 0.2 U µl Roche Protector RNase inhibitor. snRNA-seq libraries were prepared using the 10X Genomics Chromium Next GEM Single Cell 3’ Reagent Kits v.3.1 with loading of ~12,000 total nuclei per sample and sequenced on the Illumina HiSeq 4000 instrument.

The Cell Ranger software was used to perform demultiplexing and align reads to mm39. Nuclei were filtered using Seurat based on the total number of genes and molecules, and the percentage of mitochondrial reads (500 < nFeature_RNA < 6,000; 1,000 < nCount_RNA < 25,000; percent.mt < 15%). Dimensionality reduction was performed in Seurat using UMAP. Differential gene expression between clusters was performed using the FindMarkers function. Adipocyte nuclei were identified based on the expression of established markers (*Lipe*, *Plin4*, *Plin1* and *Pparg*) and filtered for reclustering using Seurat. UMAP plots of adipocyte nuclei were visualized using Loupe Browser of the imported analysis from Seurat. KEGG pathway enrichment analysis of differentially expressed genes between clusters or samples was performed using limma and biomaRt.

### Statistical analyses

Data were analysed using R (v.4.3.2) for sequencing data and Prism (v.9.0) for statistical significance of all other data. Data distribution was assumed to be normal but this was not formally tested. Results are presented as the mean ± s.e.m. for all replicates included in each analysis. No animals or data points were excluded from the analyses. Statistical tests and significance thresholds are specified in the figure legends.

### Reporting summary

Further information on research design is available in the Nature Portfolio Reporting Summary linked to this article.

## Supplementary information

Reporting Summary

Supplementary Table 1 RNA-sequencing analysis of Ndusf2-KO and Bmal1-KO fractionated adipocytes, related to Fig. 3. The *P* values are from the DESeq2 Wald test, and the adjusted *P* values are Benjamini–Hochberg false discovery rate (FDR)-corrected *P* values

Supplementary Table 2 Top 50 differentially expressed genes in adipocyte clusters, related to Fig. 3. The *P* values are from the Seurat Wilcoxon rank-sum test, and the adjusted *P* values are Benjamini–Hochberg FDR-corrected *P* values.

Supplementary Table 3 Gene and pathway lists for adipocyte clusters in control mice, related to Fig. 3. Gene-level *P* values are from the Seurat Wilcoxon rank-sum test, and pathway *P* values are from limma. Adjusted *P* values are Benjamini–Hochberg FDR-corrected *P* values.

Supplementary Table 4 Differentially expressed genes between samples, related to Fig. 3. The *P* values are from the Seurat Wilcoxon rank-sum test, and the adjusted *P* values are Benjamini–Hochberg false discovery rate FDR-corrected *P* values.

Supplementary Table 5 Primer sequences for qPCR.

## Source data

Source Data Fig. 1 Statistical source data.

Source Data Fig. 2 Statistical source data.

Source Data Fig. 3 Statistical source data.

Source Data Fig. 4 Statistical source data.

Source Data Extended Data Fig. 1 Statistical source data.

Source Data Extended Data Fig. 2 Statistical source data.

Source Data Extended Data Fig. 2 Unprocessed immunoblots.

Source Data Extended Data Fig. 3 Statistical source data.

Source Data Extended Data Fig. 3 Unprocessed immunoblots.

Source Data Extended Data Fig. 4 Statistical source data.

Source Data Extended Data Fig. 5 Statistical source data.

Source Data Extended Data Fig. 6 Statistical source data.

Source Data Extended Data Fig. 6 Unprocessed immunoblots.

Source Data Extended Data Fig. 7 Statistical source data.

Source Data Extended Data Fig. 7 Unprocessed immunoblots.

Source Data Extended Data Fig. 9 Statistical source data.

Source Data Extended Data Fig. 10 Statistical source data.

## Data Availability

The data in this study will be made publicly available according to publication date in the Gene Expression Omnibus (GEO) under accession no. GSE245850. Source data are provided with this paper.
