# Supplementary material for: Adipocyte NADH dehydrogenase reverses circadian and diet-induced metabolic syndrome
Source: Nat Metab. 2026 Feb 18;8(3):559–71. doi: 10.1038/s42255-026-01464-5 (PMC13031130; doi:10.1038/s42255-026-01464-5)
Supplement: Supplementary file 1 — Reporting Summary [file 42255_2026_1464_MOESM1_ESM.pdf]

nature portfolio

Corresponding author(s): Chelsea Hepler and Joe Bass

Last updated by author(s): Jan 5, 2026

Reporting Summary

Nature Portfolio wishes to improve the reproducibility of the work that we publish. This form provides structure for consistency and transparency in reporting. For further information on Nature Portfolio policies, see our Editorial Policies and the Editorial Policy Checklist.

Statistics

For all statistical analyses, confirm that the following items are present in the figure legend, table legend, main text, or Methods section.

n/a	Confirmed	
☐	☒	The exact sample size (*n*) for each experimental group/condition, given as a discrete number and unit of measurement
☐	☒	A statement on whether measurements were taken from distinct samples or whether the same sample was measured repeatedly
☐	☒	The statistical test(s) used AND whether they are one- or two-sided<br>*Only common tests should be described solely by name; describe more complex techniques in the Methods section.*
☐	☒	A description of all covariates tested
☐	☒	A description of any assumptions or corrections, such as tests of normality and adjustment for multiple comparisons
☐	☒	A full description of the statistical parameters including central tendency (e.g. means) or other basic estimates (e.g. regression coefficient) AND variation (e.g. standard deviation) or associated estimates of uncertainty (e.g. confidence intervals)
☐	☒	For null hypothesis testing, the test statistic (e.g. *F*, *t*, *r*) with confidence intervals, effect sizes, degrees of freedom and *P* value noted<br>*Give P values as exact values whenever suitable.*
☒	☐	For Bayesian analysis, information on the choice of priors and Markov chain Monte Carlo settings
☐	☒	For hierarchical and complex designs, identification of the appropriate level for tests and full reporting of outcomes
☒	☐	Estimates of effect sizes (e.g. Cohen's *d*, Pearson's *r*), indicating how they were calculated

Our web collection on statistics for biologists contains articles on many of the points above.

Software and code

Policy information about availability of computer code

Data collection	Metabolic cage data (food intake, respiratory exchange ratio, energy expenditure, activity) was recorded and processed using the Promethion system general data processing macro provided by Sable Systems International.
Data analysis	We used publicly-accessible software and standard parameters to analyze our data. For RNA-Seq, we used STAR (v2.5.0), DESeq2 (v1.36.0), and pathfindR (v2.3.0). For single-nuclei RNA-seq, we used Cell Ranger software (v7.2.0), Loupe Browser (v7.0.1), Seurat (v5), and limma and biomaRt (2.48.2). Data acquisition and analysis for metabolomics were carried out by Xcalibur 4.1 software and Tracefinder 4.1 software, respectively. Metabolic cage analysis data was analyzed in R (v4.1.2). Adipocyte size analysis was performed using the Keyence BZ-X Analyzer software. Western blot images were quantified with Li-Cor Image Studio Fc software (v6.0). All other analyses were performed in Graphpad Prism (v9.0).

For manuscripts utilizing custom algorithms or software that are central to the research but not yet described in published literature, software must be made available to editors and reviewers. We strongly encourage code deposition in a community repository (e.g. GitHub). See the Nature Portfolio guidelines for submitting code & software for further information.

Data

Policy information about availability of data

All manuscripts must include a data availability statement. This statement should provide the following information, where applicable:

- Accession codes, unique identifiers, or web links for publicly available datasets
- A description of any restrictions on data availability
- For clinical datasets or third party data, please ensure that the statement adheres to our policy

Data Availability: Data in this study will be made publicly available by publication date in NCBI's Gene Expression Omnibus (GEO) and are accessible through the GEO Series accession number GSE245850 (reviewer token: qtclsskyzjcdzix).

Research involving human participants, their data, or biological material

Policy information about studies with human participants or human data. See also policy information about sex, gender (identity/presentation), and sexual orientation and race, ethnicity and racism.

Reporting on sex and gender	n/a
Reporting on race, ethnicity, or other socially relevant groupings	n/a
Population characteristics	n/a
Recruitment	n/a
Ethics oversight	n/a

Note that full information on the approval of the study protocol must also be provided in the manuscript.

Field-specific reporting

Please select the one below that is the best fit for your research. If you are not sure, read the appropriate sections before making your selection.

☒ Life sciences ☐ Behavioural & social sciences ☐ Ecological, evolutionary & environmental sciences

For a reference copy of the document with all sections, see nature.com/documents/nr-reporting-summary-flat.pdf

Life sciences study design

All studies must disclose on these points even when the disclosure is negative.

Sample size	No statistical methods were applied to predetermine sample size. Sample sizes were based on prior experience with similar experimental setups.
Data exclusions	No data were excluded.
Replication	All experimental findings were reproduced with biological replicates as specified in figure legends. All additional replication attempts were successful.
Randomization	For animal studies, mice were grouped according to body weight, so that each group had the same starting average body weight.
Blinding	For LCMS runs and adipocyte size distribution analysis, experimenters were blinded to the experimental condition of each sample.

Reporting for specific materials, systems and methods

We require information from authors about some types of materials, experimental systems and methods used in many studies. Here, indicate whether each material, system or method listed is relevant to your study. If you are not sure if a list item applies to your research, read the appropriate section before selecting a response.

Materials & experimental systems

n/a	Involved in the study
☐	☒ Antibodies
☒	☐ Eukaryotic cell lines
☒	☐ Palaeontology and archaeology
☐	☒ Animals and other organisms
☒	☐ Clinical data
☒	☐ Dual use research of concern
☒	☐ Plants

Methods

n/a	Involved in the study
☒	☐ ChIP-seq
☒	☐ Flow cytometry
☒	☐ MRI-based neuroimaging

Antibodies

Antibodies used	OXPHOS cocktail (Abcam ab110413, 1:250), NDUFA9 (Abcam ab14713, 1:1000), NDUFA10 (Santa Cruz sc-376357, 1:1000), NDUFS2 (Abcam ab192022, 1:1000), UQCRC1 (Abcam ab110252, 1:1000), VDAC (Thermo PA1-954A, 1:1000), mono and dimethyl Arginine (Abcam ab412, 1:500), Blue Native OXPHOS antibody cocktail (Abcam #ab110412, 1:250), goat anti-mouse IRDye 680RD (LI-COR 926-68070, 1:10,000), goat anti-rabbit IRDye 800CW (LI-COR 926-32211, 1:10,000).
Validation	All antibodies chosen have been widely published and contain validation statements in the vendor websites.

Animals and other research organisms

Policy information about studies involving animals; ARRIVE guidelines recommended for reporting animal research, and Sex and Gender in Research

Laboratory animals	2-3 month old C57BL/6J (Jackson Laboratories #000664), mPer2Luc (Jackson Laboratories # 006852), Adiponectin-Cre (Jackson Laboratories #028020), Bmal1-fx/fx (Dr. C. Bradfield, University of Wisconsin), NDI1-LSL (Dr. N. Chandel, Northwestern University), and Ndufs2-fx/fx (Dr. J. López-Barneo, Universidad de Sevilla) mice were used. Mice were housed in light- and temperature-controlled chambers on a 12h light-dark cycle.
Wild animals	No wild animals were used in this study.
Reporting on sex	Only male mice were used in this study.
Field-collected samples	No samples were collected in the field in this study.
Ethics oversight	All animal procedures were in accordance with guidelines of the Institutional Animal Care and Use Committee at Northwestern University.

Note that full information on the approval of the study protocol must also be provided in the manuscript.

Plants

Seed stocks	n/a
Novel plant genotypes	n/a
Authentication	n/a
